# IAA-Mediated Haustorium Formation in *Phelipanche aegyptiaca*: Transcriptional Insights and Anti-Parasitic Strategies

**DOI:** 10.3390/plants14111591

**Published:** 2025-05-23

**Authors:** Xin Hu, Xiaolei Cao, Qiuyue Zhao, Xiaojian Zeng, Yingming Wei, Zhaoqun Yao, Sifeng Zhao

**Affiliations:** Key Laboratory at the Universities of Xinjiang Uygur Autonomous Region for Oasis Agricultural Pest Management and Plant Protection Resources Utilization, Agriculture College, Shihezi University, Shihezi 832003, China; huxin@stu.shzu.edu.cn (X.H.); tulanduocxl@sina.com (X.C.); zhaoqiuyue1@stu.shzu.edu.cn (Q.Z.); cengxiaojian@stu.shzu.edu.cn (X.Z.); weiyingming@stu.shzu.edu.cn (Y.W.)

**Keywords:** *Phelipanche aegyptiaca*, haustorium, indole-3-acetic acid, cell proliferation, parasitic ability

## Abstract

*Phelipanche aegyptiaca* is an obligate root-parasitic weed that parasitizes crop roots, threatening the safety of agricultural production. However, the molecular mechanisms underlying the formation of *P. aegyptiaca* haustorium remain largely unclear. Here, we employed transcriptomics to investigate the molecular events in *P. aegyptiaca* haustorium formation induced by indole-3-acetic acid. Our study revealed that during *P. aegyptiaca* haustorium formation, the cell proliferation activity at the tip of the radicle was highest during the young stage and then gradually declined. The differentially expressed genes upregulated during haustorium formation were mainly enriched in DNA replication and plant hormone signal transduction, while those that were downregulated were enriched in biosynthesis of secondary metabolites. Additionally, interfering with the auxin signal weakened the parasitic ability of *P. aegyptiaca*. These findings enhance our understanding of the mechanism of *P. aegyptiaca* haustorium formation and contribute to the targeted development of new pesticides for inhibiting *P. aegyptiaca*.

## 1. Introduction

*Phelipanche aegyptiaca* (basionym: *Orobanche aegyptiaca*) is an obligate root-holoparasitic plant [1], predominantly distributed across Africa, Asia, and the Mediterranean region [2]. *Phelipanche aegyptiaca* primarily parasitizes dicotyledonous plants and has a strong pathogenicity toward crops, leading to significant yield losses [3]. The management of *P. aegyptiaca* is complicated by the fact that its life cycle is predominantly completed underground [3]. *Phelipanche aegyptiaca* represents a serious threat to local agricultural security, resulting in a significant yield reduction and severely compromising agricultural safety [4,5,6,7,8,9]. In Xinjiang, China, melon cultivation is severely impacted by *P. aegyptiaca*, with an annual infestation area ranging from 3500 to 5000 hm^2^. Among these, approximately 1500–2000 hm^2^ experience total yield loss due to intensive parasitism. Additionally, *P. aegyptiaca* infestation affects around 7000 hm^2^ of processing tomato fields, resulting in yield reductions of 30–80% [4,6].

During evolution, certain taxa within the Orobanchaceae family completely lost their photosynthetic capacity, and their root systems have undergone degradation [10]. Consequently, these plants are unable to grow independently of their hosts and rely on host plants for water, organic carbon, and nitrogen [11,12,13,14]. These parasitic plants attach to the roots of host plants through a specialized organ, the haustorium, and invade the host vascular system [15]. After the *P. aegyptiaca* haustorium attaches to the host, it successfully establishes a physical connection with the host, mediating the direction of water and nutrient transport [16,17]. In obligate parasitic plants, when the seed germinates, a terminal haustorium develops at the tip of the radicle, halting root growth and changing the cell elongation direction from longitudinal to radial [18,19,20]. The development of the haustorium structure commences with swelling of the root tip due to a change in the direction of cell division [21]. In the obligate holoparasitic plant *P. aegyptiaca*, which lacks chlorophyll, haustorium development is characterized by swelling at the root tip. This is jointly driven by the elongation of apical cells and the polar protrusion of epidermal cells [21]. The final structure formed is a short-cell elongation structure called the papillae [22]. Papillae produce adhesive substances that fix the haustorium to the host root [23,24]. This special structure serves as the basis for the successful invasion of host plants by *P. aegyptiaca*.

The formation of the haustorium structure is triggered by stimulation with host-derived chemical substances known as haustorium-inducing factors (HIFs) [14]. Indole-3-acetic acid (IAA) has been identified as an HIF for *P. aegyptiaca*, and it can induce the differentiation of haustorium structures at *P. aegyptiaca* radicle tips [25,26]. The auxin-biosynthesis gene *YUC3*, which encodes flavin-containing monooxygenase, is upregulated in haustorial cells of the hemiparasitic plant *Phtheirospermum japonicum* [27]. The auxin-signaling network mediates xylem bridge formation in *Phtheirospermum japonicum* [28]. The competitive auxin antagonist *p*-chlorophenoxyisobutyric acid (PCIB) reduces haustorium formation in hemiparasitic plant *Triphysaria versicolor* [29]. These studies indicate that the auxin-signaling pathway is involved in the haustorium formation process of parasitic plants. However, the molecular events underlying HIF-induced haustorium formation in the holoparasitic plant *P. aegyptiaca* remain largely unknown.

This study aimed to systematically elucidate the role of IAA in haustorium formation in *P. aegyptiaca* by investigating its dynamic processes through an integrated approach combining cell proliferation analysis, transcriptomics, and validation of anti-parasitic strategies. The findings will advance the understanding of haustorium organogenesis in parasitic plants and provide a theoretical foundation for the development of environmentally friendly control strategies targeting auxin signaling pathways.

## 2. Materials and Methods

### 2.1. Plant Materials and Growth Conditions

*Phelipanche aegyptiaca* seeds were collected from tomato fields in Tacheng, Xinjiang, China, where the crop was infested by this parasite. The seeds were surface sterilized via immersion in a 2% sodium hypochlorite solution for 15 min, followed by a 3-min soak in 75% ethanol and thorough rinsing with sterile water three times. Subsequently, sterile *P. aegyptiaca* seeds were transferred to 24-well plates and incubated in sterile water for 2 days to break dormancy. The seeds were then treated with a 0.1 μM GR24 solution (Macklin Biochemical Co., Ltd., Shanghai, China) for 3–4 days to induce germination [26,30]. After germination, they were treated with 0.1 mM IAA (Macklin Biochemical Co., Ltd., Shanghai, China) to trigger haustorium formation [25]. All experiments were carried out in the dark at 25 °C. At 0, 1, 3, 5, and 7 days post-treatment with 0.1 mM IAA for haustorium induction, the diameters of *P. aegyptiaca* radicles under different treatments were measured and recorded, and the obtained data were used to quantify the developmental progress of *P. aegyptiaca* haustorium.

### 2.2. 5-Ethynyl-2′-Deoxyuridine (EdU) Staining

IAA-treated *P. aegyptiaca* samples collected at 1, 3, and 7 days post-treatment from the aforementioned experimental groups were processed. The three sample groups treated with IAA for different durations were immersed in an incubation solution containing 10 μM EdU (Beyotime Biotechnology Co., Ltd., Shanghai, China) and incubated in the dark at 25 °C for 2 h. The samples were collected, rinsed with phosphate-buffered saline, and fixed with 4% paraformaldehyde for 4 h, and 5–7 μm paraffin sections were prepared using a microtome. EdU was labeled with IF555 to show red fluorescence, and 4′,6-diamidino-2-phenylindole (DAPI) was used to stain the nuclei, showing blue fluorescence. The sections were viewed under an upright fluorescence microscope (NIKON ECLIPSE C1) to collect fluorescence images. The total intensity of the red EdU fluorescence signal within the overlapping area of the red EdU fluorescence and the blue DAPI-stained nuclei fluorescence was measured using ImageJ (V1.54g) software in the red fluorescence channel. The number of DAPI-labeled nuclei was counted. The average nuclear fluorescence intensity of EdU was utilized to quantify the EdU signal in the tissues of *P. aegyptiaca* under different treatment conditions, and to evaluate the impact of IAA on its cell proliferation activity.

The average nuclear fluorescence intensity of EdU was calculated as follows:

Average nuclear fluorescence intensity of EdU = Total intensity of EdU fluorescence signal/Number of DAPI-labeled nuclei

### 2.3. RNA Sequencing

*Phelipanche aegyptiaca* seeds were treated according to the above-mentioned methods until the germination stage. The germinated *P. aegyptiaca* radicles were subjected to the following treatments: an untreated control group (T0) and treatment with 0.1 mM IAA for 1 (T1) and 3 days (T3). Samples were collected at the corresponding time points for each treatment. There were three biological replicates for this experiment. The collected samples were extracted using the ethanol precipitation method with CTAB-PBIOZOL reagent and Trizol reagent. Successfully extracted RNA was dissolved in 50 μL of DEPC-treated ddH2O, and total RNA was identified and quantitatively analyzed using a Qubit fluorometer (Thermo Fisher Scientific, Waltham, MA, USA) and a Qsep400 high-throughput bio-fragment analyzer (BiOptic Inc., New Taipei, Taiwan).

The mRNA was enriched with oligo (dT) magnetic beads. After fragmentation, double-stranded cDNA was synthesized via reverse transcription. Steps such as end-repair were completed, and sequencing adapters were ligated. A library with an insert size of 250–350 bp was constructed using magnetic bead purification and fragment screening. After PCR amplification, magnetic bead purification was performed again, and the product was dissolved. Precise quality control and quantitative analysis were carried out after library construction.

Based on the effective concentration of the library and the requirements for the target amount of sequencing data, the libraries were pooled and sequenced on an Illumina sequencer (NovaSeq 6000, Illumina, San Diego, CA, USA). cDNA library construction was followed by sequencing, which was carried out on an Illumina sequencing platform by Metware Biotechnology Co., Ltd. (Wuhan, China).

Raw sequencing data were filtered using fastp (V0.23.2); paired-end reads were discarded if either read contained > 10% undetermined nucleotides (N) or if > 50% of bases exhibited low-quality scores (Q ≤ 20). All downstream analyses utilized the resultant high-fidelity clean reads. The filtered clean reads were aligned to the publicly reported *P. aegyptiaca* reference genome [10] for subsequent analysis.

### 2.4. Identification and Functional Enrichment Analysis of DEGs

To quantitatively assess the gene expression levels, featureCounts (V2.0.3) was employed to compute the gene alignment metrics. Subsequently, the fragments per kilobase of exon per million reads mapped (FPKM) values for individual genes were calculated, factoring in gene length and serving as a reliable measure of gene expression. In the gene heatmap generated from FPKM data after Z-score normalization, a value of zero indicates that the gene’s expression level equals the mean across all samples.

DESeq2 (V1.22.1) was utilized to perform pairwise differential expression analysis between the two experimental groups. Differentially expressed genes (DEGs) were identified based on a rigorous set of criteria: |log2 fold change| ≥ 1 and a false discovery rate (FDR) < 0.05.

Functional enrichment analyses were carried out on the identified DEGs using hypergeometric tests. For Kyoto Encyclopedia of Genes and Genomes (KEGG) pathway enrichment analysis, hypergeometric distribution tests were conducted, with pathways as the fundamental units. For Gene Ontology (GO) enrichment analysis, enrichment calculations were executed using GO terms.

### 2.5. qRT-PCR

*Phelipanche aegyptiaca* samples were processed and underwent RNA extraction using methods identical to those employed for RNA sequencing. Using the extracted total RNA as a template, cDNA was synthesized using a reverse transcription kit (TOLOBIO, Shanghai, China). The 20-μL quantitative real-time PCR (qRT-PCR) reaction system contained the fluorescent dye SYBR Green I (TOLOBIO, Shanghai, China), an appropriate amount of cDNA template, and specific primers. qRT-PCR was conducted on an Applied Biosystems 7500 Real Time PCR System following the reagent manufacturer’s protocol. The thermal cycling protocol comprised initial denaturation at 95.0 °C for 30 s, followed by 40 cycles of two-step amplification, with denaturation at 95.0 °C for 10 s and combined annealing/extension at 60.0 °C for 30 s. The gene-specific primer sequences are listed in Supplementary Table S1. The 2^−ΔΔCt^ method was used to analyze the RT-qPCR data, with *actin* (*act1*) as the internal reference gene [31]. Three independent replicated experiments were performed.

### 2.6. Greenhouse Experiment

When the seedlings of melon cultivar K1076 (a susceptible variety) [32] grew to the two-leaf stage, they were transplanted into pots with soil and inoculated with *P. aegyptiaca* seeds. The experimental design included three treatment groups: the 1 mM IAA aqueous solution treatment group, the 15 μM auxin activity inhibitor *p*-chlorophenoxyisobutyric acid (PCIB) [29] (Macklin Biochemical Co., Ltd., Shanghai, China) aqueous solution treatment group, and the control group irrigated with only water. The treated groups were irrigated three times in the early, middle, and late stages after transplantation and watered regularly during this period. The control group received irrigation with only water throughout the growth cycle.

Two months after transplant, total parasitism by *P. aegyptiaca* and parasitism at each developmental stage under the three treatments were recorded. The muskmelon plant height was measured. The experiment was conducted in a growth chamber at 25 °C with a 16-/8-h light/dark photoperiod. To ensure the reliability of the experimental data, each experiment was repeated three times, and all flowerpots were arranged according to a randomized block design.

### 2.7. Statistical Analysis

Statistical analyses were performed using IBM SPSS Statistics software (version 26.0; IBM Corp., Armonk, NY, USA). Continuous variables are presented as the mean ± standard deviation (SD). For intergroup comparisons, parametric assumptions were verified through Shapiro–Wilk normality testing and Levene’s homogeneity of variance assessment. Independent two-tailed Student’s *t*-tests were employed for pairwise comparisons between two experimental groups. When comparing three or more groups under single-factor conditions, one-way analysis of variance (ANOVA) was conducted, followed by Tukey’s honestly significant difference (HSD) post hoc test for multiple comparisons. The threshold for statistical significance was set at *p* = 0.05 for all inferential analyses.

## 3. Results

### 3.1. Temporal Dynamics of Haustorium Development in P. aegyptiaca Induced by IAA

**Figure 1:**
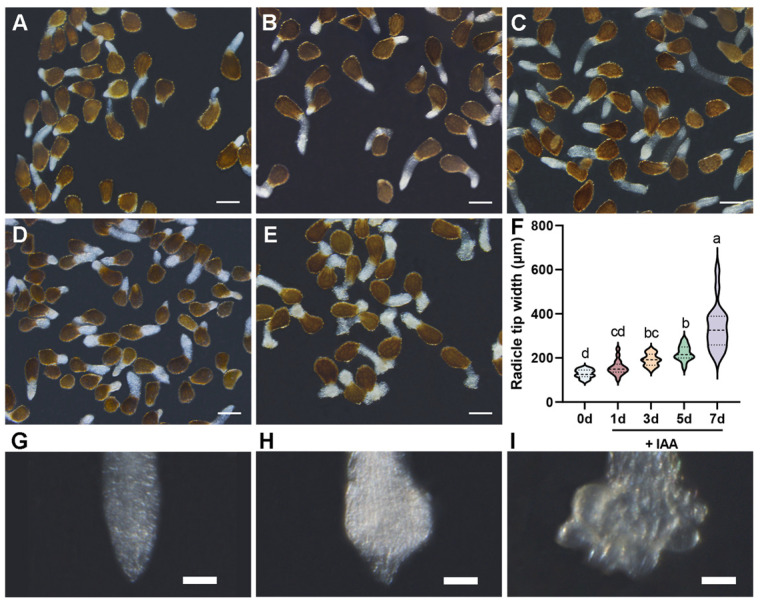
The characteristics of the time-line formation of *Phelipanche aegyptiaca* haustorium induced by indole-3-acetic acid (IAA). (**A**) Germinated *P. aegyptiaca* seeds without IAA application (0 d). (**B**–**E**) Germinated *P. aegyptiaca* seeds treated with 0.1 mM IAA for 1, 3, 5, and 7 days, respectively. Scale bar: 500 μm. (**F**) Maximum width of the *P. aegyptiaca* radicle tip in (**A**–**E**). Different letters indicate statistically significant differences (*p* < 0.05). (**G**–**I**) The radicle tip of *P. aegyptiaca* at the young stage, expansion stage, and differentiation stage, respectively. Scale bar: 100 μm.

The radicles of *P. aegyptiaca* seeds experience different developmental stages as they develop into the haustorium stage. We treated germinated *P. aegyptiaca* seeds with 0.1 mM IAA and continuously observed the morphological changes in the radicles after treatment. Treatment without IAA was used as the control group to explore the characteristic changes during *P. aegyptiaca* haustorium formation. Under IAA treatment, the maximum width of the radicle tip exhibited progressive enlargement with prolonged treatment (Figure 1) [26]. On the first day after induction, no significant difference was detected between IAA-treated (156.3 ± 33.92 μm) and the control radicles (128.1 ± 19.14 μm) (Figure 1A,B,F,G). However, by day three post-induction, IAA-treated radicles showed a marked expansion in width (191.5 ± 27.73 μm; *p* < 0.05), which was significantly larger than that of the control group (Figure 1A,C,F). During this period, the *P. aegyptiaca* radicle tip expanded (Figure 1H). This growth pattern became more pronounced over time, with the width reaching 221.5 ± 34.29 μm at day five post-induction (Figure 1A,D,F) and 339.8 ± 103.4 μm by day seven (Figure 1A,E,F), and during this period, a differentiated haustorium formed at the *P. aegyptiaca* radicle tip (Figure 1I). Our results depicted the timeline for IAA-mediated *P. aegyptiaca* haustorium formation. The early stage (1–3 d) was characterized by initial radial expansion, and the late stage (5–7 d) was marked by continuous tissue hypertrophy and further differentiation, leading to the formation of an irregular multicellular structure, namely the haustorium (Figure 1I), at the *P. aegyptiaca* radicle tip.

### 3.2. Spatiotemporal Dynamics of P. aegyptiaca Radicles at Different Developmental Stages

**Figure 2:**
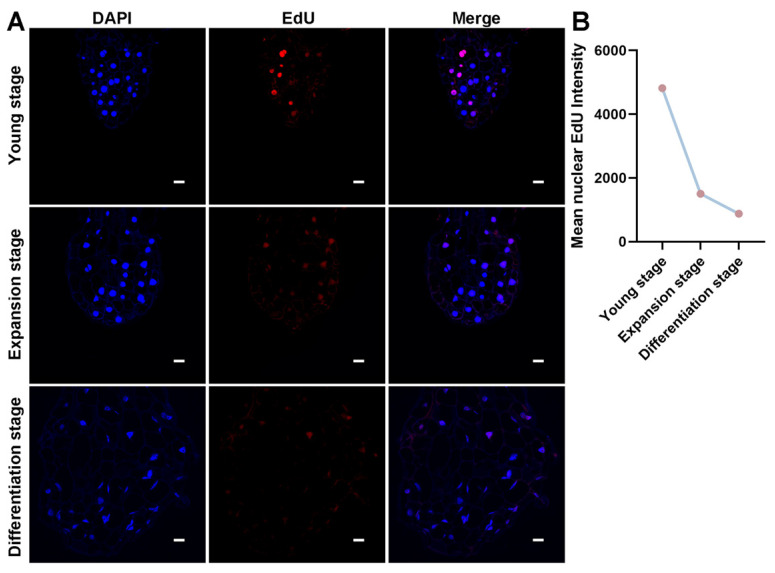
Different haustorium development stages of *Phelipanche aegyptiaca* via EdU staining. (**A**) Nuclear signals of cell nuclei labeled by DAPI, nuclear signals of proliferating cell nuclei labeled by EdU, and superimposed images of the two signals in the three developmental stages of haustorium formation in *P. aegyptiaca* radicle cells: young stage, expansion stage, and differentiation stage. Scale bar: 20 μm. (**B**) Variation trend of the average nuclear fluorescence intensity of EdU in *P. aegyptiaca* radicle cells during the three haustorium development stages.

To investigate the spatiotemporal dynamics of cell proliferation during *P. aegyptiaca* haustorium development, EdU fluorescence labeling was performed on *P. aegyptiaca* specimens at three key haustorium development stages. These three stages were the young stage (one day after IAA treatment), the expansion stage (three days after IAA treatment), and the haustorium stage (seven days after IAA treatment). Quantitative analysis of EdU fluorescence showed that the cell proliferation activity at the *P. aegyptiaca* radicle tip was the highest at the young stage and then gradually decreased (Figure 2B). At the young stage, the radicles exhibited vigorous cell proliferation, with an average EdU nuclear fluorescence intensity of 4816, indicating active DNA synthesis in the early haustorium initiation stage. The average EdU nuclear fluorescence intensity in the radicles at the swelling stage was 1500, with a 68.9% decrease in fluorescence intensity, suggesting that cell proliferation gradually decreased as the cells transitioned to the expansion stage. When the radicles differentiated into the haustorium stage, the average EdU nuclear fluorescence intensity was 879, accounting for 18.3% of the initial value, with a weak fluorescence signal.

The spatial localization of the EdU signal further confirmed the dynamic changes in the proliferative region at the *P. aegyptiaca* radicle tip. In image analysis, the Merge column visually displayed the spatial distribution pattern of EdU-positive cells (cells in a proliferative state) within the entire cell population by superimposing the blue fluorescence image of DAPI-labeled nuclei and the red fluorescence image of EdU-labeled proliferating cells. In the young stage, proliferating cells were mainly concentrated in the apical meristem region of the radicle (Figure 2A). In the expansion and differentiation stages, the proliferating cells showed a characteristic distribution pattern in which they migrated basally. This phenomenon might be closely related to the haustorium developmental process, suggesting a transition from cell proliferation activity to functional specialization (Figure 2A). These results indicate that during *P. aegyptiaca* haustorium development, the cell proliferation rate decreased over time, suggesting that haustorium development gradually approached the mature stage.

### 3.3. Differential Expression Genes in Three P. aegyptiaca Haustorium Development Stages

**Figure 3:**
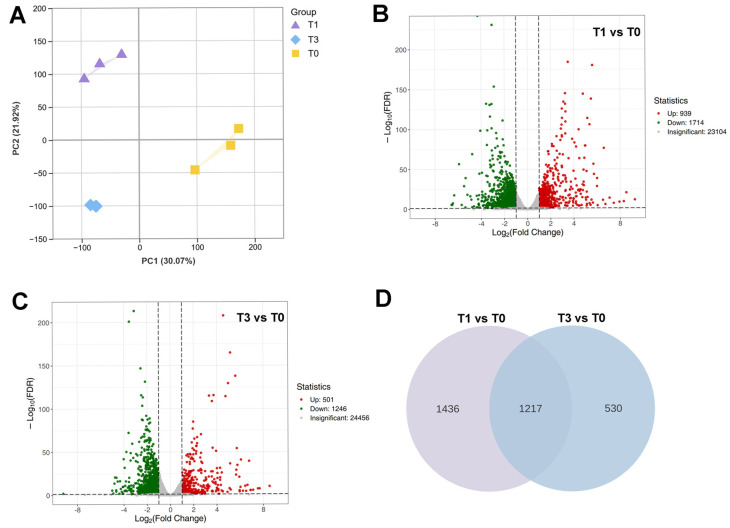
Analysis of DEGs in three *Phelipanche aegyptiaca* haustorium development stages. (**A**) Principal component analysis of all samples. Sample groups are distinguished by color, and individual samples are represented as dots. (**B**,**C**) The numbers of upregulated and downregulated DEGs in T1 vs. T0 and T3 vs. T0, respectively. (**D**) Venn diagram of DEGs in the pairwise comparisons of T1 vs. T0 and T3 vs. T0.

To further explore the molecular regulatory mechanisms governing the initiation of *P. aegyptiaca* haustorium development, we performed RNA sequencing on *P. aegyptiaca* radicles under three treatments: the untreated control group (T0), one-day IAA treatment (T1), and three-day IAA treatment (T3). Principal component analysis (PCA) demonstrated significant clustering among T0, T1, and T3 (Figure 3A). In the T1 vs. T0 comparison, 2653 DEGs were identified, among which 939 were upregulated and 1714 were downregulated (Figure 3B). In the T3 vs. T0 comparison, 1747 DEGs were identified, with 501 upregulated and 1246 downregulated (Figure 3C). From T1 to T3, the number of upregulated DEGs decreased by 47%. There were 1217 common DEGs between T1 vs. T0 and T3 vs. T0 (Figure 3D).

### 3.4. GO and KEGG Enrichment Analyses of DEGs in P. aegyptiaca Haustorium Development Stages

**Figure 4:**
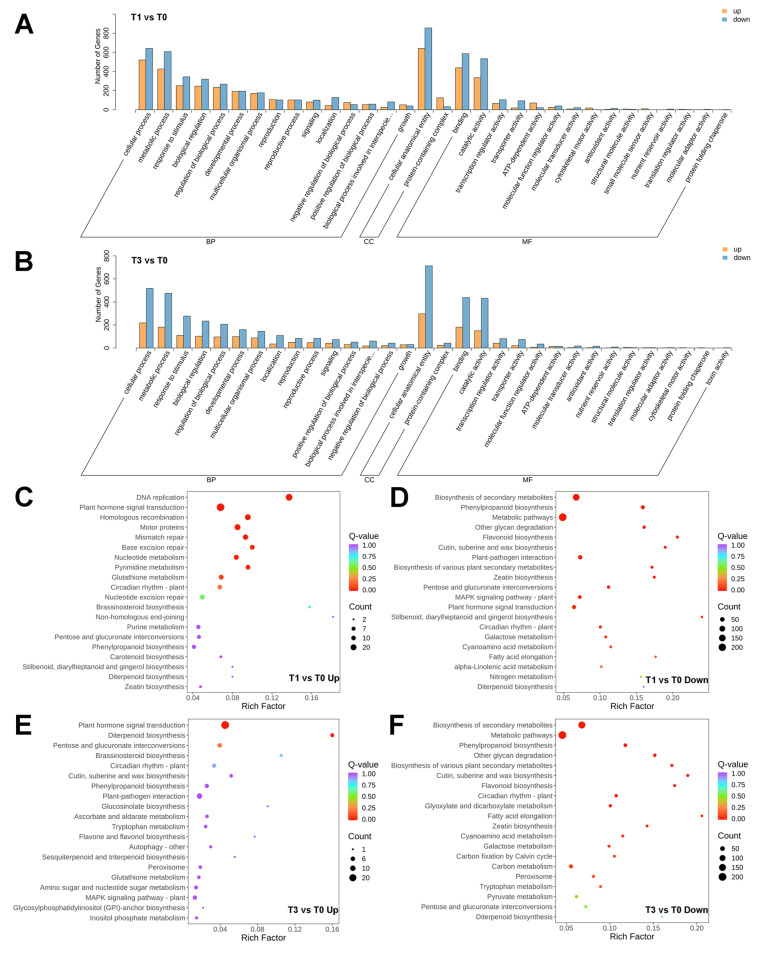
Enrichment analysis of DEGs. (**A**,**B**) GO enrichment analysis of up- and down-regulated DEGs in T1 vs. T0 and T3 vs. T0, respectively. (**C**–**F**) Top 20 enriched pathways in the KEGG enrichment analysis of upregulated DEGs in T1 vs. T0, downregulated DEGs in T1 vs. T0, upregulated DEGs in T3 vs. T0, and downregulated DEGs in T3 vs. T0, respectively.

During haustorium development, IAA treatment induced dynamic functional reprogramming in biological processes (BPs), cellular components (CCs), and molecular functions (MFs) (Figure 4A,B). One day after IAA treatment (T1 vs. T0), cellular processes and metabolic processes were highly enriched among BP terms, reflecting the rapid activation of cell proliferation and energy metabolism. CC terms were related to the remodeling of cellular anatomical entities, and binding and catalytic activity dominated MFs, emphasizing the roles of protein interactions and enzymatic reactions in early auxin signaling (Figure 4A, Supplementary Table S2). With continued IAA treatment (T3 vs. T0), BP terms remained enriched in cellular processes and metabolic processes, but the degree of upregulation decreased. The sustained enrichment of cellular anatomical entities corresponds with morphological specialization, and binding and catalytic activity continued to be the core aspects of MFs (Figure 4B, Supplementary Table S3).

KEGG enrichment analysis of the early response to IAA (T1 vs. T0) in melon showed that upregulated DEGs were significantly enriched in DNA replication and plant hormone signaling pathways (Figure 4C, Supplementary Table S4). This indicates strong activation of cell cycle processes and hormonal signal interactions on the first day of IAA treatment. Conversely, downregulated DEGs exhibited significant suppression of biosynthesis of secondary metabolites and phenylpropanoid biosynthesis (Figure 4D, Supplementary Table S5), suggesting a trade-off between growth promotion and defensive metabolite production. KEGG enrichment analysis after continued IAA treatment (T3 vs. T0) revealed that upregulated DEGs remained enriched in plant hormone signaling pathways, although the DNA replication pathway was no longer enriched (Figure 4E, Supplementary Table S6). Downregulated DEGs exhibited more extensive suppression of biosynthesis of secondary metabolites and metabolic pathways, highlighting distinct pathway characteristics compared to T1 (Figure 4F, Supplementary Table S7).

### 3.5. Analysis of Key Pathways Based on KEGG Enrichment Results

**Figure 5:**
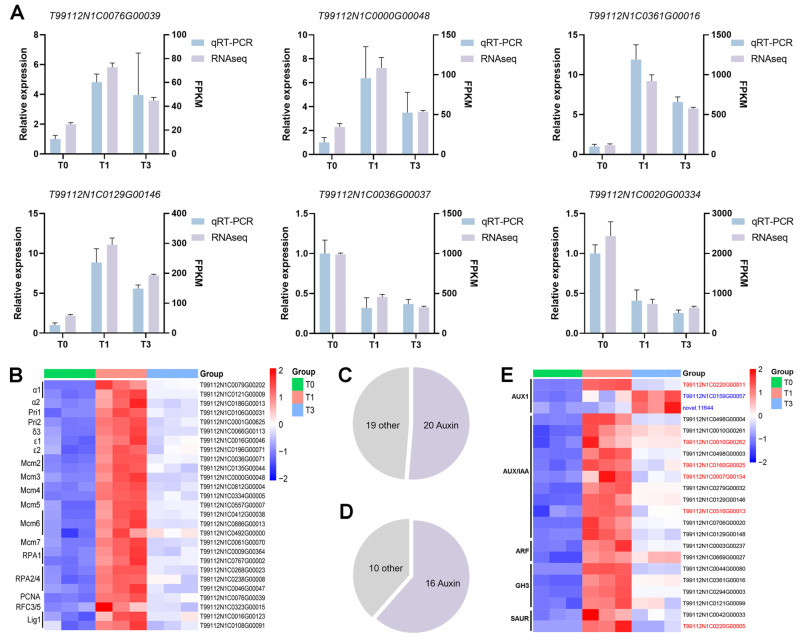
Analysis of key pathways based on KEGG enrichment results. (**A**) Consistency assessment between RNA sequencing and qRT-PCR results for DEGs in key signaling pathways. (**B**) Heatmap analysis of 27 DEGs in the replication complex pathway enriched in the DNA replication pathway by upregulated DEGs in T1 vs. T0. (**C**,**D**) Proportion of DEGs in the auxin signal transduction pathway, which is enriched by upregulated DEGs in the plant hormone signal transduction pathway in T1 vs. T0 and T3 vs. T0, respectively. (**E**) Heatmap analysis of auxin signaling pathway genes enriched in upregulated DEGs from both T1 vs. T0 and T3 vs. T0. Shared DEGs are labeled in black, while T1-specific and T3-specific DEGs are marked in red and blue, respectively.

The expression levels of genes in key KEGG pathways, namely six DEGs involved in DNA replication (T99112N1C0076G00039, T99112N1C0000G00048), plant hormone signal transduction (T99112N1C0361G00016, T99112N1C0129G00146), and biosynthesis of secondary metabolites (T99112N1C0036G00037, T99112N1C0020G00334), were verified using qRT-PCR. The qRT-PCR results were highly consistent with the trends in the transcriptome data (Figure 5A), indicating the reliability of the expression patterns of these genes in the key pathways predicted by KEGG analysis.

Among the 28 DEGs in the DNA replication pathway that were upregulated in T1 vs. T0, 27 were enriched in the replication complex pathway (Figure 5B), including genes related to the minichromosome maintenance (MCM) helicase complex, such as *Mcm2*–*Mcm7*, genes related to the DNA polymerase α-primase complex, such as *α1*, *α2*, *Pri1*, and *Pri1*, and genes related to single-stranded DNA-binding protein (RPA), such as *RPA1* and *RPA2/4*. This indicates that IAA widely activated the replication complex pathway in *P. aegyptiaca* at one day after induction, but these genes were no longer significantly enriched at three days after induction. In both T1 vs. T0 and T3 vs. T0, upregulated DEGs were significantly enriched in the plant hormone signal transduction pathway. Further classification and statistical analysis of the DEGs enriched in the plant hormone signal transduction pathway in T1 vs. T0 and T3 vs. T0 revealed that the highest proportion of genes was enriched in the auxin signal transduction pathway. Among the 39 DEGs in T1 vs. T0, 20 were related to auxin, and among the 26 DEGs in T3 vs. T0, 16 were related to auxin (Figure 5C,D). These activated genes encompass key components of the auxin signaling pathway, including AUX1 transporters, AUX/IAA repressors, ARF transcription factors, and downstream *GH3* and *SAUR* family genes (Figure 5E) [33]. This indicates that the auxin signal transduction pathway plays a dominant role in *P. aegyptiaca* haustorium development.

### 3.6. Auxin Signaling Regulates the Parasitic Capacity of P. aegyptiaca

**Figure 6:**
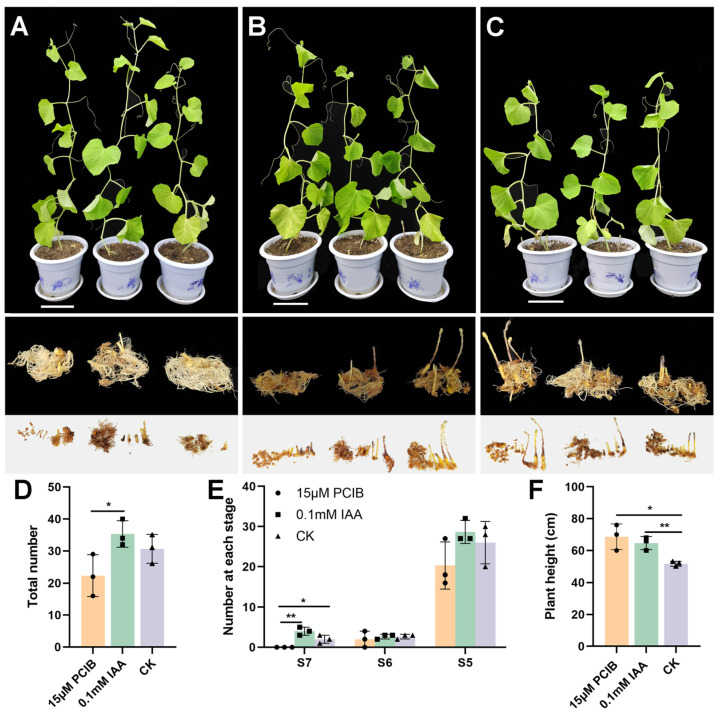
Effects of IAA and auxin inhibitor on the parasitic ability of *Phelipanche aegyptiaca*. (**A**–**C**) Growth status of melon seedlings inoculated with *P. aegyptiaca*, parasitic damage to their root systems, and developmental status of *P. aegyptiaca* under treatments with 15 μM PCIB, 0.1 mM IAA, and water (CK), respectively. (**D**) Total number of *P. aegyptiaca* attachments to the host roots in the three treatments (* *p* < 0.05). (**E**) Number of *P. aegyptiaca* attachments to the host roots at different developmental stages in the three treatments (* *p* < 0.05; ** *p* < 0.01). S7: *P. aegyptiaca* emergence; S6: *P. aegyptiaca* develops a young shoot > 2 cm but has not emerged; S5: Adventitious roots form at the nodules and connect with other roots < 1 cm. (**F**) Plant heights of the host plants under the three treatments (* *p* < 0.05; ** *p* < 0.01).

To evaluate the role of auxin signaling in the *P. aegyptiaca* parasitism process, we treated *P. aegyptiaca*-infected susceptible melons with 15 μM of the auxin inhibitor PCIB [29], 0.1 mM IAA, and water (control group). There were differences in the degree of damage to the hosts infected with *P. aegyptiaca* under different treatments. The total number of *P. aegyptiaca* attachments on the roots of the hosts treated with PCIB was 22.3 ± 6.51, which was significantly reduced compared to 35.3 ± 4.2 in the IAA-treated group (*p* < 0.05, Figure 6A–D). Although the number in the PCIB-treated group was not significantly different from 30.7 ± 4.5 in the control group, it also decreased to a certain extent (Figure 6A–D). Further classification of *P. aegyptiaca* at different stages showed the absence of *P. aegyptiaca* at the emergence stage (Stage S7) in the PCIB-treated group. At stage S7, *P. aegyptiaca* parasitism in the PCIB-treated group exhibited a significant reduction compared to that in the IAA-treated group (4 ± 1) and the control group (2 ± 1) (*p* < 0.05; Figure 6A–C,E). This indicates that PCIB application weakens *P. aegyptiaca*’s parasitic ability, hinders *P. aegyptiaca* development, and delays *P. aegyptiaca* emergence. In addition, the roots of IAA-treated hosts were severely yellowed and showed an unhealthy state compared to the other treatments (Figure 6B). The plant height was 51.7 ± 1.5 cm in the control group (Figure 6C,F). The plant height of the PCIB-treated group was the highest, reaching 68.7 ± 8.1 cm (*p* < 0.05), and that of the IAA-treated group was at an intermediate level, being 64.7 ± 4.2 cm (*p* < 0.01) (Figure 6A–C,F).

## 4. Discussion

### 4.1. IAA-Driven P. aegyptiaca Haustorium Development

In this study, through dynamic observations of IAA-induced *P. aegyptiaca* haustorium development, we found that the width of the radicle gradually increased with the extension of the treatment duration. It was significantly larger than that of the control group after three days, and the haustorium differentiated after seven days, showing the maximum width. This indicates that IAA promotes *P. aegyptiaca* radicle growth and haustorium formation, which is consistent with previous research showing that auxin promotes plant organ development [25,34]. Notably, the turning point at three days after induction indicates a crucial transition from induction to active growth, which is consistent with previous reports on auxin-regulated cell wall relaxation and meristem activation in parasitic plants [33,35,36]. These results suggest that IAA is a potent inducer of *P. aegyptiaca* haustorium morphogenesis. Radial expansion starting at three days after induction emphasizes the role of IAA in driving parasitic adaptation by changing the root architecture.

### 4.2. Cell Proliferative Characteristics During P. aegyptiaca Haustorium Development

During *P. aegyptiaca* haustorium development, the radicle experienced three typical developmental stages: young, expansion, and haustorium-differentiation stages. EdU-labeling experiments showed that the cell proliferation activity at the radicle tip was the highest during the young stage and then gradually decreased, and it was the lowest during the haustorium-differentiation stage. This is consistent with the slowing of the cell proliferation rate during haustorium morphogenesis at the radicle tip in the hemiparasitic plant *Striga hermonthica* [19]. This indicates that, during the early stage, cell proliferation is active, providing a sufficient number of cells for the initiation and initial development of the haustorium. In the haustorium-differentiation stage, the cell proliferation activity weakens, and cells become more involved in differentiation and specialization to form a haustorium structure with specific functions. This dynamic change in cell proliferation and differentiation is consistent with previously reported results showing that auxin is involved in regulating the timely termination of plant organ meristems [37], further demonstrating the important role of IAA in regulating *P. aegyptiaca* haustorium formation. As the development of the *P. aegyptiaca* haustorium progresses, the cell proliferation rate decreases, indicating that the haustorium is gradually approaching maturity.

### 4.3. Transcriptional Insights into P. aegyptiaca Haustorium Development

Early IAA response (T1 vs. T0): the enrichment of the DNA replication pathway corroborates the observed active cell proliferation in young *P. aegyptiaca* radicles (Figure 2), indicating a substantial demand for DNA synthesis during early haustorium development. This pathway includes genes encoding 10 MCM helicase complexes [38,39], five genes associated with the DNA polymerase α-primase complex [40] and five genes for single-stranded DNA binding proteins [41], all of which are critical for initiation, unwinding, and extension at the replication fork. The upregulation of replication components activates molecular mechanisms that support rapid cell proliferation, consistent with the GO enrichment results for cellular processes. IAA systematically activates the complete auxin signaling cascade in *P. aegyptiaca*, demonstrating that the endogenous auxin response machinery serves as a molecular basis for haustorium formation (Figure 5E). This finding challenges the prevailing paradigm that primarily focuses on auxin transport-mediated establishment of host-parasite interactions [18,19].

Sustained IAA response (T3 vs. T0): the loss of significance in the DNA replication pathway corresponds to a 58% reduction in the number of upregulated DEGs within the cellular processes GO term and a decrease in EdU signal intensity, reflecting diminished cell proliferation activity. The activation of the auxin signaling pathway is less pronounced compared to that in the T1 stage, yet the entire pathway remains actively engaged. Elucidating the regulatory association between auxin signaling modules and haustorium formation in *P. aegyptiaca* in future research may provide new theoretical references for the development of control strategies against parasitic plants.

Downregulation of secondary metabolite biosynthesis-related DEGs in both stages suggests resource prioritization for *P. aegyptiaca* haustorium development under IAA induction.

### 4.4. Anti-Parasitic Strategies Against P. aegyptiaca

Inhibiting the auxin signaling pathway with PCIB effectively reduced the parasitism of *P. aegyptiaca*. This result is consistent with previous studies, which demonstrated that the disruption of auxin signaling affects the parasitic capability of *Phtheirospermum japonicum* on the host *Arabidopsis thaliana* [28], and that the application of PCIB to the roots of *Triphysaria versicolor* reduces haustorium formation [29]. The auxin distribution pattern in the radicle of *Striga hermonthica* is critical for haustorium formation [19]. Tryptophan and its metabolite IAA inhibit radicle elongation in *Orobanche minor* [42]. It further validates the important role of IAA in the development and parasitism of *P. aegyptiaca* haustorium. PCIB enhanced host growth, emphasizing the metabolic cost of parasitism. Therefore, control strategies that target the auxin signaling pathway, such as using auxin inhibitors or interfering with the expression of related signaling molecules, should be developed to inhibit the formation and parasitism of *P. aegyptiaca* haustorium, thus, controlling *P. aegyptiaca*. Although this study reveals the critical regulatory role of IAA signaling in haustorium development of *P. aegyptiaca*, the key effector genes governing haustorium formation remain to be elucidated. In future research, we will endeavor to identify or develop highly specific inhibitors targeting *Phelipanche* species to avoid potential impacts on the environment or host crops. Additionally, the field stability of auxin inhibitors and their effects on non-target organisms (such as aquatic life, other plant species, and human systems) require comprehensive evaluation through ecotoxicological assessments [43]. 

## 5. Conclusions

This study elucidates the pivotal regulatory role of IAA in *P. aegyptiaca* haustorium formation. Key findings include: (1) Radicle tip cell proliferation activity peaks during early haustorium development and subsequently declines (2) Upregulated DEGs are significantly enriched in DNA replication and plant hormone signal transduction pathways during early haustorium development; and (3) Auxin signaling disruption effectively impairs parasitic capability. These results provide novel insights into the molecular mechanisms of haustorial organogenesis in parasitic plants and establish a theoretical foundation for developing eco-friendly control strategies targeting auxin signaling pathways.

## Data Availability

The raw transcriptome data have been deposited in the National Center for Biotechnology Information (NCBI) database under accession number PRJNA1236295.

## Supplementary Materials

The following supporting information can be downloaded at: https://www.mdpi.com/article/10.3390/plants14111591/s1, Table S1: Gene-specific primers for real time quantitative PCR; Table S2: GO enrichment analysis of DEGs in the comparison of T1 vs. T0; Table S3: GO enrichment analysis of DEGs in the comparison of T3 vs. T0; Table S4: KEGG enrichment analysis of up-regulated DEGs in the comparison of T1 vs. T0; Table S5: KEGG enrichment analysis of down-regulated DEGs in the comparison of T1 vs. T0; Table S6: KEGG enrichment analysis of up-regulated DEGs in the comparison of T3 vs. T0; Table S7: KEGG enrichment analysis of down-regulated DEGs in the comparison of T3 vs. T0.
